# Eruptive acral lentiginosis following chemotherapy for acute lymphoblastic leukemia: A case series

**DOI:** 10.1002/ccr3.5015

**Published:** 2021-10-28

**Authors:** Cathal O’Connor, Clodagh Ryan, Lesley‐Ann Murphy

**Affiliations:** ^1^ Department of Dermatology South Infirmary Victoria University Hospital Cork Ireland; ^2^ Department of Paediatrics and Child Health University College Cork Cork Ireland; ^3^ Department of Paediatric Haematology Mercy University Hospital Cork Ireland

**Keywords:** acral lentigines, chemotherapy, leukemia

## Abstract

Lentigines are brown macules which develop due to increased proliferation of melanocytes at the dermo‐epidermal junction. We report three cases of acral lentiginosis in children following chemotherapy for acute lymphoblastic leukaemia (ALL) which have persisted following cessation of chemotherapy, despite avid photoprotection. Generalised eruptive naevi with subsequent development of dysplastic naevi and melanoma in situ have been reported following chemotherapy, highlighting the importance of continued clinical observation.

## Graphical abstract

Children may develop acral lentiginosis following chemotherapy for acute lymphoblastic leukemia. Subsequent development of dysplasia and melanoma in situ has been reported in these lesions, highlighting the need for clinical surveillance.

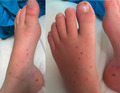

## 1 INTRODUCTION

Lentigines are brown macules which develop due to increased proliferation of melanocytes at the dermo‐epidermal junction. They commonly occur in healthy people but can also be seen in genodermatoses such as Noonan syndrome with multiple lentigines. Eruptive lentigines have previously been described in the context of inflammatory dermatoses,^1^ phototherapy,^2^ and immunomodulatory therapy.^3^ Acral eruptive lentigines have been described following chemotherapy^4^ or as a paraneoplastic phenomenon.^5^ We report three cases of acral lentiginosis in children following chemotherapy for acute lymphoblastic leukemia (ALL) which have persisted following cessation of chemotherapy, despite avid photoprotection. All patients remain in remission from ALL.

## 2 CASE SERIES

**FIGURE 1:**
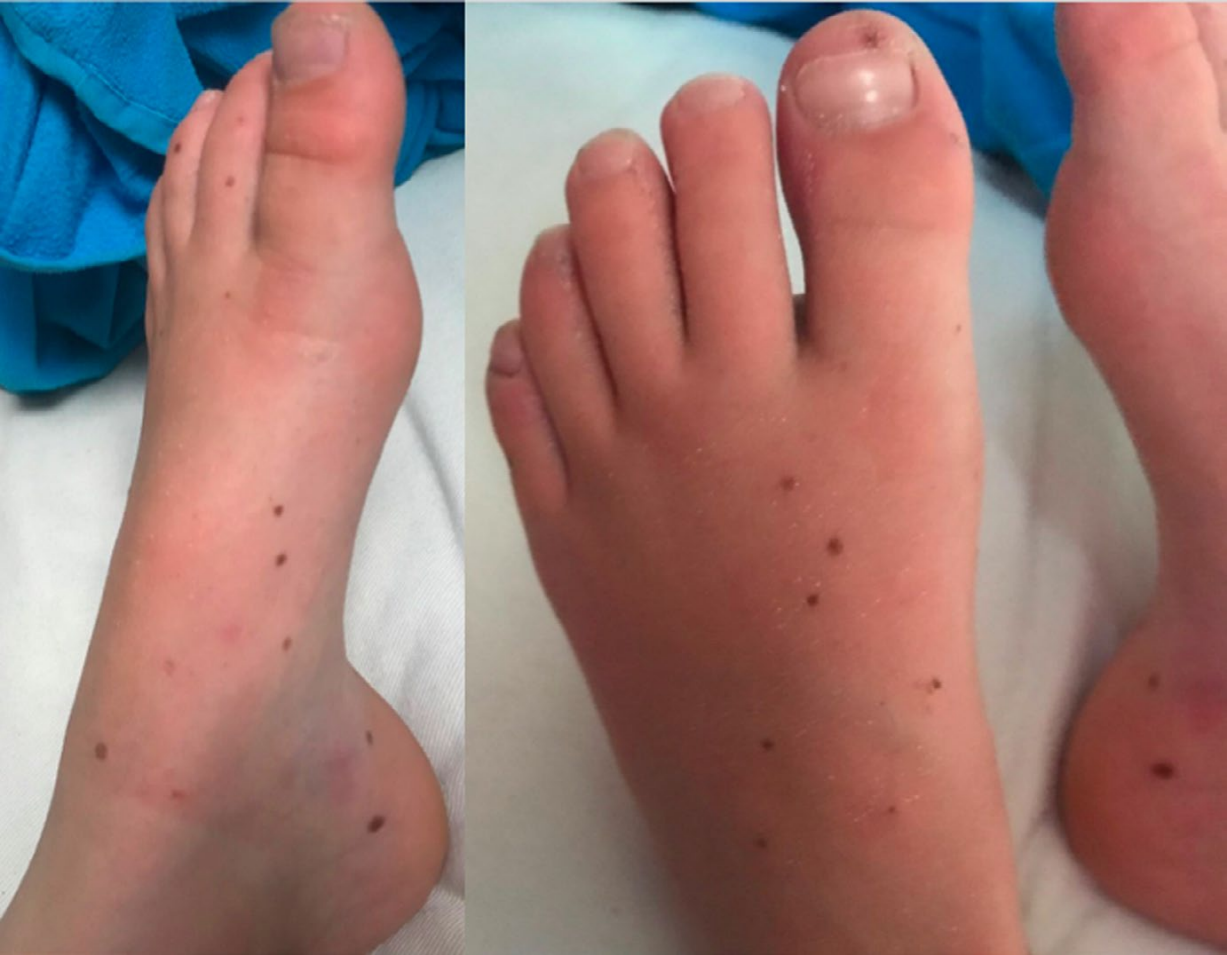
Multiple lentigines on the dorsal surfaces of the feet

Patient one was a five‐year‐old Caucasian boy diagnosed with pre‐B ALL in 2013 and treated as per the UKALL 2011 protocol regimen A. This regimen includes dexamethasone, vincristine, pegaspargase, methotrexate, mercaptopurine, doxorubicin, cyclophosphamide, and cytarabine. Following cessation of chemotherapy in 2016, multiple small brown macules were noted on the palmar and plantar surfaces of hands and feet (Figure 1). These have persisted for over 4 years.

**FIGURE 2:**
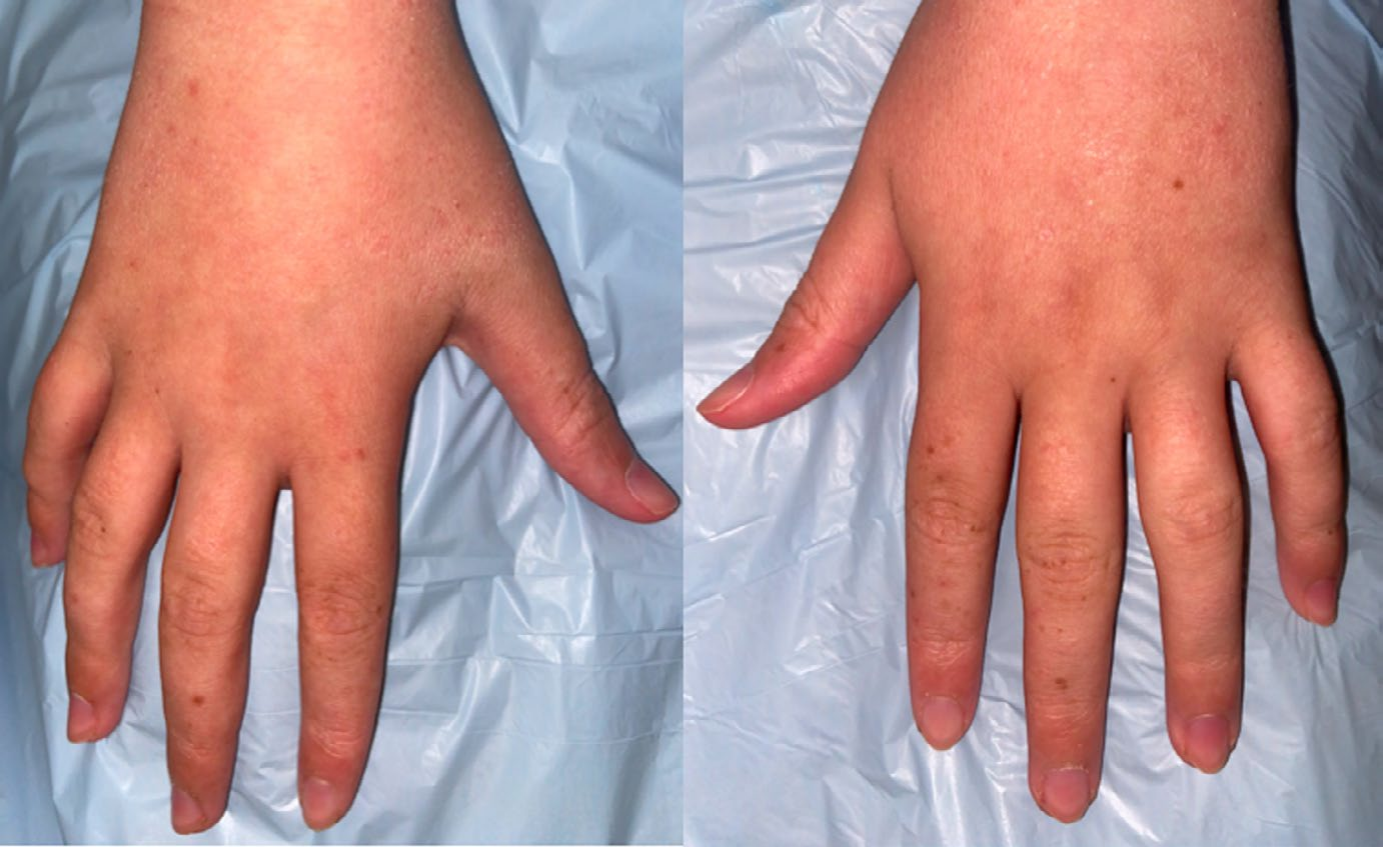
Multiple lentigines on the dorsal surfaces of the hands

Patient two was a nine‐year‐old Caucasian boy diagnosed with pre‐B ALL in 2017 and treated as per the COG ALL 1131 protocol. This regimen includes prednisolone, vincristine, pegaspargase, methotrexate, mercaptopurine, anthracyclines, cyclophosphamide, and cytarabine. During maintenance chemotherapy in 2020, multiple small brown macules were noted on the dorsal surfaces of fingers and feet (Figure 2).

**FIGURE 3:**
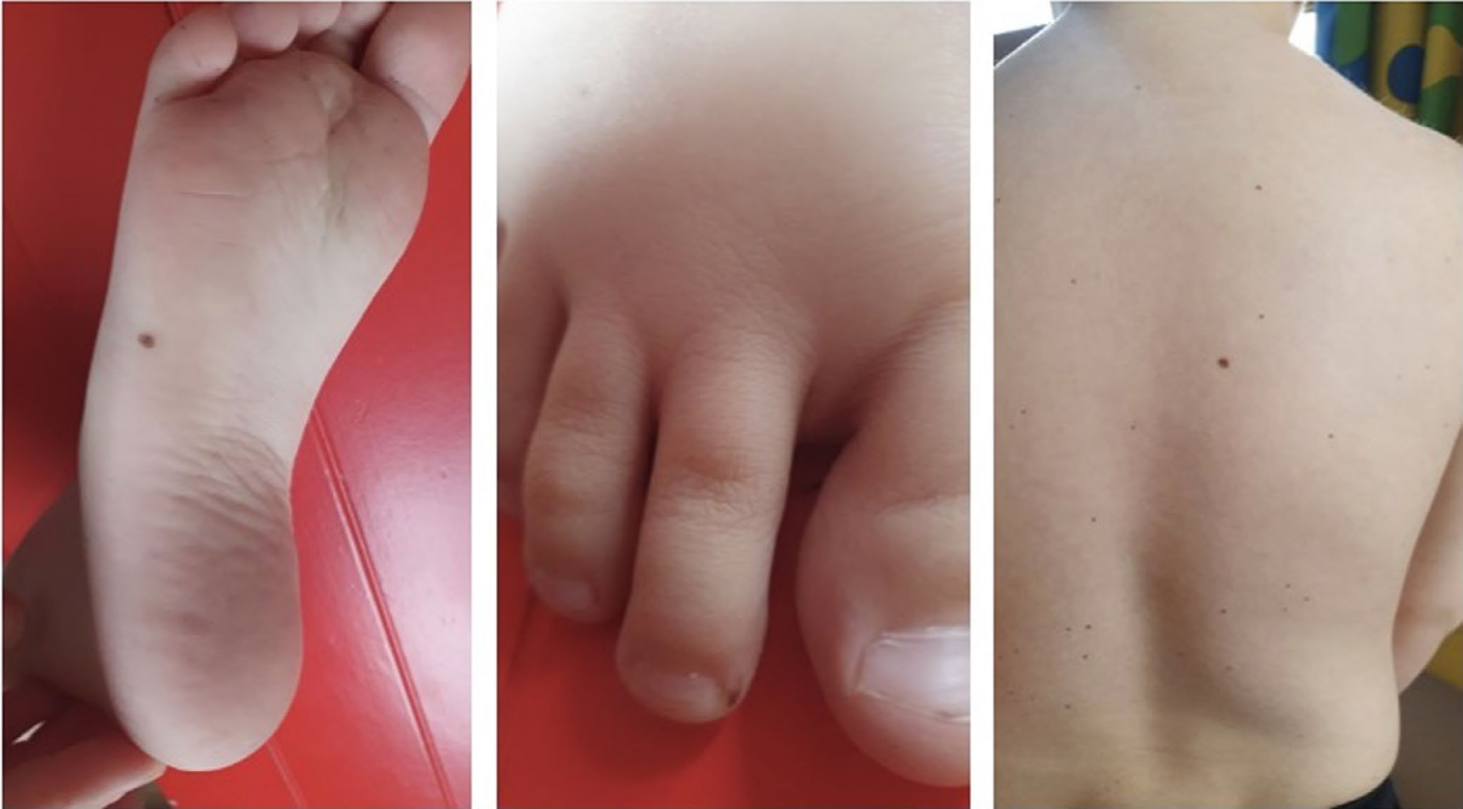
Multiple lentigines on the feet and trunk

Patient three was a seven‐year‐old Caucasian boy diagnosed with pre‐B ALL in 2018 and treated as per the UKALL 2011 protocol regimen A. During maintenance chemotherapy in 2020, multiple small brown macules were noted on the palmar and plantar surfaces of hands and feet, as well as extensive macules on the trunk (Figure 3).

## 3 DISCUSSION

Lentigines are the most basic form of melanocytic proliferation, on a spectrum that progresses to junctional, compound, and dermal nevi. Inflammation or immunosuppression can induce melanocyte hyperplasia. Cytotoxic agents may also induce lentigines via modulation of tumor‐specific lymphocytes.^4^ Eruptive lentigines have been described in the context of psoriasis, atopic dermatitis, and drug eruptions, as well as secondary to both psoralen and ultraviolet A (PUVA) and ultraviolet B (UVB) phototherapy. Topical tacrolimus and topical immunotherapy with dibutyl squaric acid have also been associated with lentigines. Drugs implicated in eruptive lentiginosis include immunomodulatory therapy such as methotrexate, azathioprine, apremilast, etanercept, adalimumab, infliximab, ustekinumab, secukimumab, and ixekizumab, and cancer chemotherapy.

Acral lentiginosis is characterized by eruptive lentigines limited to the hands and feet. The predilection for acral sites may occur due to a combination of altered immunosurveillance and elevated local trophic factors present in acral skin.^6^ Exposure to ultraviolet radiation may facilitate their development. Eruptive acral lentiginosis has been reported following chemotherapy for ALL in children,^7^ and following treatment with capecitabine^4^ and tegafur^8^ (both prodrugs of 5‐fluorouracil).

There are obvious difficulties in ascertaining the culprit drug for lentiginosis when multiple cytotoxic and immunosuppressive drugs are administered concomitantly as a part of a chemotherapeutic protocol. Anecdotally, we have also noted several cases of eruptive acral lentiginosis in patients treated with high dose cytarabine for acute myeloid leukemia (C Ryan, personal observation). Development of lentigines may depend on a complex milieu of cancer, immunosuppression, cytotoxic drug therapy, and UV exposure.

Generalized eruptive nevi with subsequent development of dysplastic nevi and melanoma in situ have been reported following chemotherapy,^9^ highlighting the importance of continued clinical observation.

## CONFLICT OF INTEREST

None declared.

## AUTHOR CONTRIBUTIONS

LAM suggested publication of the case series. COC reviewed all patients and wrote the initial draft. COC, CR, and LAM reviewed and edited the manuscript.

## CONSENT

Consent for publication was provided by the parents of the children in this case series.
